# The Roles of Non-Coding RNAs in Radiotherapy of Gastrointestinal Carcinoma

**DOI:** 10.3389/fcell.2022.862563

**Published:** 2022-04-20

**Authors:** Jie Li, Juan Sun, Zhen Liu, Ziyang Zeng, Siwen Ouyang, Zimu Zhang, Mingwei Ma, Weiming Kang

**Affiliations:** Department of General Surgery, Peking Union Medical College Hospital, Chinese Academy of Medical Sciences & Peking Union Medical College, Beijing, China

**Keywords:** non-coding RNAs, radiotherapy, colorectal cancer, rectal cancer, gastric cancer

## Abstract

Radiotherapy (RT), or radiation therapy, has been widely used in clinical practice for the treatment of local advanced gastrointestinal carcinoma. RT causes DNA double-strand breaks leading to cell cytotoxicity and indirectly damages tumor cells by activating downstream genes. Non-coding RNA (including microRNAs, long non-coding RNAs (ncRNAs), and circular RNAs) is a type of RNA that does not encode a protein. As the field of ncRNAs increasingly expands, new complex roles have gradually emerged for ncRNAs in RT. It has been shown that ncRNAs can act as radiosensitivity regulators in gastrointestinal carcinoma by affecting DNA damage repair, cell cycle arrest, irradiation-induced apoptosis, cell autophagy, stemness, EMT, and cell pyroptosis. Here, we review the complex roles of ncRNAs in RT and gastrointestinal carcinoma. We also discuss the potential clinical significance and predictive value of ncRNAs in response to RT for guiding the individualized treatment of patients. This review can serve as a guide for the application of ncRNAs as radiosensitivity enhancers, radioresistance inducers, and predictors of response in RT of gastrointestinal carcinoma.

## Background

Gastrointestinal carcinoma poses a significant burden for human health, according to the Global Cancer Statistics 2020 (Sung et al., 2021). Gastrointestinal carcinomas can be classified as gastric cancer (GC), colon cancer (CC), and rectal cancer (RC). At the time of diagnosis, most patients are diagnosed with advanced-stage cancer due to the lack of characteristic symptoms and effective screening methods (Dekker et al., 2019; Smyth et al., 2020). Although the survival rate of patients with gastrointestinal carcinoma recently improved due to the advances in treatments, the long-term survival of advanced-stage cancer patients is still poor (Shitara and Ohtsu, 2016; Biller and Schrag, 2021). Radiotherapy (RT) is an essential tool for treating patients with local advanced gastrointestinal carcinoma (Zhang et al., 2018a; Thompson et al., 2018; Tam and Wu, 2019), and the response to RT is critical to the long-term survival of these patients. Previous studies have demonstrated that various factors, including non-coding RNAs, could affect the effectiveness of RT (Grassberger et al., 2019; Ozpiskin et al., 2019; McLaughlin et al., 2020).

The ncRNAs, including microRNAs (miRNAs), long ncRNAs (lncRNAs), and circular RNAs (circRNAs), cannot encode proteins. Several reports have shown that ncRNAs can play key roles in cell cycle transition, apoptosis, metastasis, autophagy, stemness, and pyroptosis in gastrointestinal carcinoma at post-transcriptional process (Zhang et al., 2018b; Li et al., 2019a; Wang et al., 2019a; Peng et al., 2019; Li et al., 2020a; Li et al., 2020b; Yang et al., 2020; Li et al., 2021a; Peng et al., 2021; Wang et al., 2021). ncRNAs can regulate radiosensitivity by targeting mRNAs or proteins (Wang et al., 2014; Afshar et al., 2018; Chen et al., 2019; Chen et al., 2020; Gao et al., 2021). In addition, the aberrant profiles of ncRNAs in tissues or body fluids can be used as biomarkers to predict the response to RT in gastrointestinal cancer patients, guiding the selection of the treatment (Azizian et al., 2015; Campayo et al., 2018; D’Angelo et al., 2016; Kelley et al., 2017).

Previous studies have investigated whether ncRNAs participate in radiosensitivity or radioresistance and whether they are positive or negative biomarkers to predict complete response to RT. In this review, we elaborate on the roles of ncRNAs in RT and gastrointestinal carcinoma as follows: 1) ncRNAs as radiosensitivity enhancers in RT, 2) the mechanism of ncRNAs as radiosensitivity enhancers in RT, 3) ncRNAs as radioresistance inducers in RT, 4) the mechanism of ncRNAs as radioresistance inducers in RT, 5) ncRNAs as biomarkers to predict the response to RT, and 6) the clinical application of ncRNAs in gastrointestinal carcinoma. This review highlights the diverse functions of ncRNAs in RT and gastrointestinal cancer and their importance in predicting the efficacy of RT in patients with gastrointestinal cancer.

### Non-Coding RNAs Enhance Radiosensitivity

Studies have shown that tumor cell radiosensitivity is closely associated with alterations in the tumor microenvironment (TME), epigenetics, and the expression of key genes (Zhang et al., 2019a; Buckley et al., 2020; Zhang et al., 2020; Chen et al., 2021). The role of miRNAs, a type of ncRNAs, have been widely investigated in RT and gastrointestinal carcinoma as direct or indirect targets. Saeid et al. identified that miR-185 strengthened radiosensitivity by promoting irradiation-induced apoptosis of colorectal cancer (CRC) cells (Afshar et al., 2018). miR-451 were downregulated in GC and CRC samples compared to adjacent normal tissues, while overexpressed miR-451 increased the sensitivity of GC and CRC cells (Bandres et al., 2009). Ge et al. reported that miR-122-5p was increased in the plasma of patients after irradiation, and upregulated miR-122-5p strengthened the radiosensitivity by repressing cell survival and accelerating irradiation-induced apoptosis of Human Intestinal Epithelial Crypt (HIEC) cells (Ge et al., 2020). Levels of miR-130a were decreased in a resistant RC cell line and increased in a sensitive RC cell line. miR-130a sensitized the RC cells to RT via suppressing the epithelial-mesenchymal transition (EMT) and invasion (Ha Thi et al., 2019). Ji et al. found that miR-15b was significantly reduced in CRC tissues, and increased miR-15b enhanced the sensitivity of CRC cells to RT by suppressing cell growth and metastasis (Ji et al., 2018). Through a series of functional experiments, Liao et al. reported that overexpressed miR-506-3p or miR-140-5p significantly improved the radiosensitivity of CRC cells (Liao et al., 2020). miR-124 was reduced in both CRC tissues and cell lines, and elevated miR-124 improved the sensitivity of CRC cells to RT (Zhang et al., 2014; Lin et al., 2016). miR-214, miR-21-5p, and miR-519b-3p were increased in the tissues of CRC, RC, and locally advanced RC (LARC) patients that responded to RT. Besides, miR-214 enhanced the sensitivity of CRC cells to RT by repressing irradiation-induced autophagy *in vitro* and *in vivo* (Hu et al., 2018). In SW480 cells, overexpressed miR-21-5p increased the sensitivity to RT (Lopes-Ramos et al., 2014). miR-519b-3p reinforced the sensitivity of CRC cells to RT by facilitating irradiation-induced apoptosis (Luo et al., 2018). Luu et al. (2013) revealed that inhibition of let-7a repressed the sensitivity to RT in CRC cells with wild-type TP53 by negatively regulating K-Ras activity. miR-451a was elevated in the tissues of RC patients with partial response to RT, and its overexpression improved the radiosensitivity of CRC cells by repressing cell growth and reducing cell survival (Ruhl et al., 2018). Using microarray analysis and qPCR, miR-320a, miR-132, and let-7g were found to be downregulated in radioresistant cell lines, while overexpressed miR-320a, miR-132, and let-7g significantly promoted the radiosensitivity of CRC cells (Salendo et al., 2013). In addition, let-7e was reported to enhance the radiosensitivity of CRC cells by suppressing cell cycle transition and cell survival and accelerating irradiation-induced apoptosis (Samadi et al., 2019). Similarly, miR-196b strengthened the sensitivity of GC cells to RT by suppressing cell cycle transition and DNA damage repair (Shen et al., 2018). miR-320 was downregulated in both CC tissues and cell lines, and elevated miR-320 reinforced the radiosensitivity of CC cells (Wan et al., 2015). miR-100 was downregulated in CRC tissues, while overexpressed miR-100 significantly promoted the radiosensitivity of CRC cells by facilitating irradiation-induced apoptosis and suppressing DNA damage repair (Yang et al., 2015). miR-630 was decreased in the radioresistant CRC cell lines after irradiation. Upregulated miR-630 increased the sensitivity and radiation-induced cytotoxicity of CRC cells to RT (Zhang et al., 2015). miR-145 enhanced the radiosensitivity of CRC cells by antagonizing SNAI1-mediated stemness (Zhu et al., 2018).

In addition, novel emerging functions of lncRNAs and circRNAs in RT and gastrointestinal carcinoma are gradually being unveiled. Lnc-p21 levels were decreased in both GC and CRC tissues and cell lines, and elevated lnc-p21 improved the sensitivity of GC cells and CRC cells to RT (Wang et al., 2014; Chen et al., 2019). Lnc-OIP5-AS1 was downregulated in radioresistant CRC cell lines using microarray analysis and qPCR, while overexpressed lnc-OIP5-AS1 significantly promoted the radiosensitivity in CRC cells (Zou et al., 2018). Lnc-NEAT1 increased the sensitivity of CRC cells to RT by accelerating the irradiation-induced pyroptosis (Su et al., 2021). Upregulated circ-CBL.11 boosted the sensitivity of CRC cells to RT via suppressing the cell growth *in vitro* (Li et al., 2019b). The abovementioned data indicated that the ncRNAs effectively improved the sensitivity of gastrointestinal carcinoma cells to RT (Table 1).

**TABLE 1:** The radiosensitivity enhancement of non-coding RNAs in gastrointestinal carcinoma.

Cancer type	Non-coding RNAs	Expression	Sources	Sample number	Targets	Biological functions	Upstream	References
CRC	miR-185	—	—	—	IGF1R and IGF2	Promote irradiation-induced apoptosis	—	Afshar et al. (2018)
GC	lnc-p21	Decreased	Tissue and cell line	40 paired	—	Suppress cell growth, cell cycle transition, migration, and sensitize cell to RT	Irradiation	Chen et al. (2019)
CRC	lnc-p21	Decreased	Tissue and cell line	30 paired	—	Promote irradiation-induced apoptosis and enhance radiosensitivity	Irradiation	Wang et al. (2014)
CRC	miR-451	Decreased	Tissue	12 paired	MIF	Reduce cell proliferation and sensitize cell to RT	—	Bandres et al. (2009)
GC	miR-451	Decreased	Tissue	67 (45 for Kaplan-Meier analysis)	MIF	Reduce cell proliferation and sensitize cell to RT	—	Bandres et al. (2009)
RC	miR-122-5p	Increased	Serum and mice tissue	3 RC patients and 20 mice	CCAR1	Inhibit cell survival, enhance radiosensitivity, and increase cell apoptosis	Irradiation	Ge et al. (2020)
RC	miR-130a	Increased	Radiosensitive RC cells	—	SOX4	Inhibit EMT, invasion, repair of DNA damage and enhance radiosensitivity	—	Ha Thi et al. (2019)
CRC	miR-15b	Decreased	Tissue	135 paired	DCLK1	Inhibit cell growth, invasion, and metastasis and enhance radiosensitivity	—	Ji et al. (2018)
CRC	miR-506-3p and miR-140-5p	Increased	Serum	18	—	Decrease cell proliferation, survival rate, and enhance radiosensitivity	—	Liao et al. (2020)
CRC	miR-124	Decreased	Tissue and cell line	24 paired	PRRX1	Promote irradiation-induced apoptosis, inhibit EMT and cell stemness, and enhance radiosensitivity	—	Zhang et al. (2014), Lin et al. (2016)
CRC	miR-214	Decreased	Serum and cell line	10	ATG12	Inhibit IR-induced autophagy and enhance radiosensitivity	—	Hu et al. (2018)
RC	miR-21-5p	Increased	Tissue	43	SATB1	Enhance radiosensitivity	—	Lopes-Ramos et al. (2014)
RC	miR-519b-3p	Increased	Tissue	55	ARID4B	Inhibit cell growth, promote irradiation-induced apoptosis, and enhance radiosensitivity	—	Luo et al. (2018)
CRC	Let-7a	—	—	—	—	Inhibit cell growth and enhance radiosensitivity	—	Luu et al. (2013)
RC	miR-451a	Increased	Tissue	12	CAB39 and EMSY	Inhibit cell proliferation, attenuate surviving fraction, and enhance radiosensitivity	Irradiation	Ruhl et al. (2018)
CRC	miR-320a, miR-132 and let-7g	—	—	—	—	Enhance radiosensitivity	—	Salendo et al. (2013)
CRC	let‐7e	—	—	—	IGF‐1R	Arrest cell cycle transition, promote apoptosis, and enhance radiosensitivity	—	Samadi et al. (2019)
GC	miR-196b	Decreased	Cell line	—	RAD23B	Impair DNA damage repair, arrest cell cycle transition, and enhance radiosensitivity	Irradiation	Shen et al. (2018)
CC	miR-320	Decreased	Tissue and cell line	55 paired	FOXM1	Inhibit cell growth, cell cycle transition, migration, invasion, and enhance radiosensitivity	—	Wan et al. (2015)
CRC	miR-100	Decreased	Tissue and cell line	30 paired	—	Promote irradiation-induced apoptosis and DNA double-strand breaks, and enhance radiosensitivity	—	Yang et al. (2015)
CRC	miR-630	Decreased	Cell line	—	BCL2L2 and TP53RK	Enhance irradiation-induced cytotoxicity and enhance radiosensitivity	CREB	Zhang et al. (2015)
CRC	miR-145	decreased	Cell line	—	—	inhibit cell stemness and enhance radiosensitivity	SNAI1	Zhu et al. (2018)
CRC	lnc-OIP5-AS1	Decreased	Cell line	—	miR-369-3p/DYRK1A	Impair cell clonogenic survival, promote irradiation-induced apoptosis, and enhance radiosensitivity	—	Zou et al. (2018)
CRC	lnc-NEAT1	Increased	Cell line	—	miR-448/GSDME	Promote IR-induced pyroptosis and enhance radiosensitivity	Irradiation	Su et al. (2021)
CRC	circ-CBL.11	Increased	Cell line	—	miR-6778-5p/YWHAE	Suppress cell proliferation	Irradiation	Li et al. (2019b)

CRC, colorectal cancer; CC, colon cancer; GC, gastric cancer; RT, radiation therapy; RC, rectal cancer; EMT, Epithelial-Mesenchymal Transition.

### The Mechanism of Radiosensitivity Enhancement

**FIGURE 1:**
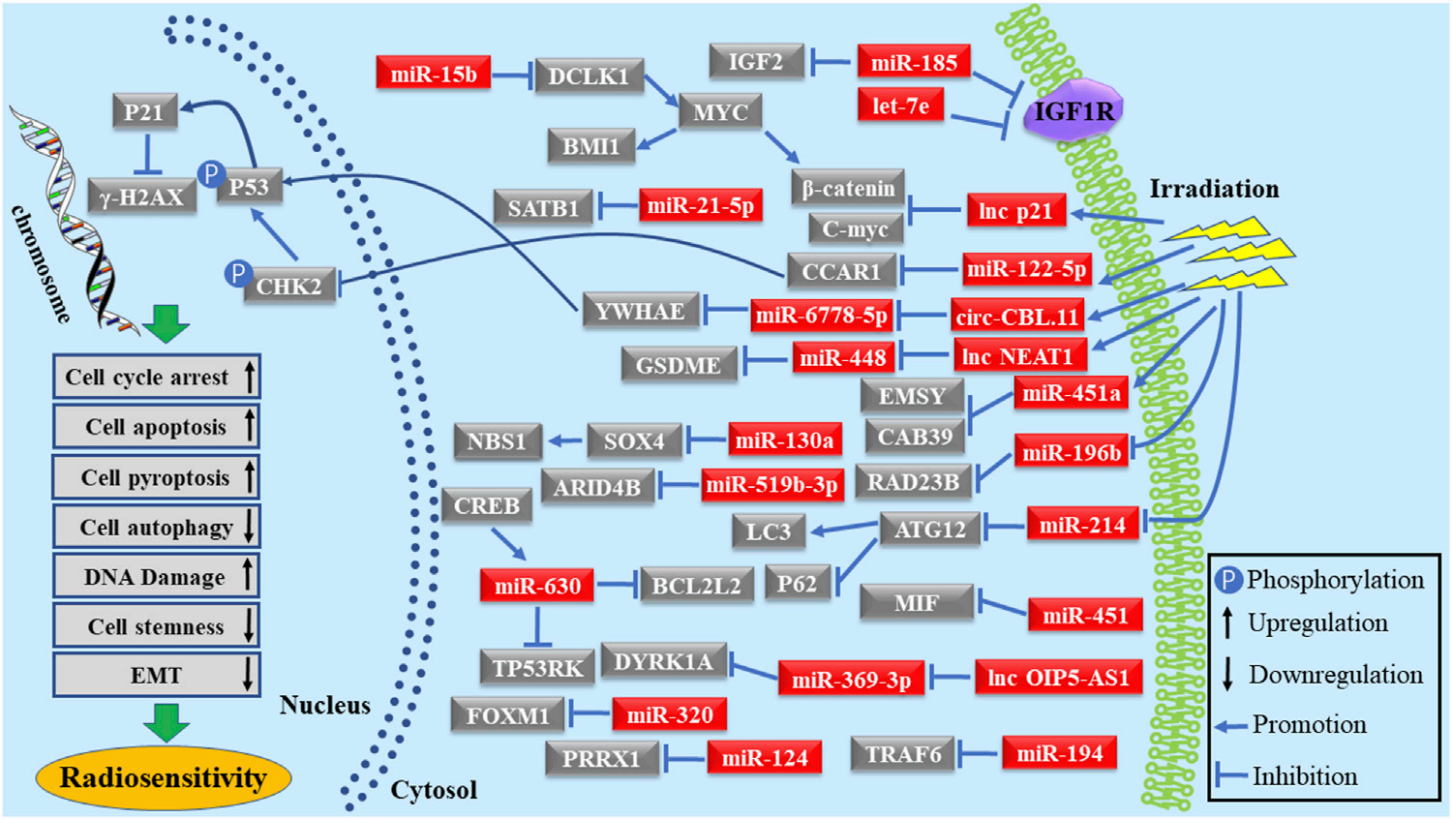
Schematic diagram of ncRNAs as radiosensitivity enhancers in gastrointestinal carcinoma.

RT directly leads to DNA damage, mainly caused by double-strand breaks in tumor cells, and indirectly damages tumor cells through the generated reactive oxygen species (De Ruysscher et al., 2019; Martin and Martin, 2020). Furthermore, radiation modifies the TME, affecting the anti-tumor immune response (Horsman et al., 2012; Grassberger et al., 2019; Shu et al., 2021). In these processes, it is possible to enhance radiation sensitivity by enhancing the transcription of specific genes or the activity of key proteins (El Bezawy et al., 2019; Ma et al., 2019). miRNAs regulate the cellular protein expression by binding to the 3′ untranslated region of mRNA, resulting in a decrease or degradation of the target genes, thus affecting the sensitivity of tumor cells to RT. Irradiation causes DNA damage and apoptosis of tumor cells; to compensate the damage, some genes are activated, triggering DNA damage repair and irradiation-induced apoptosis. It has been found that miR-185, let-7e, miR-451, miR-122-5p, miR-130a, miR-124, miR-519b-3p, miR-451a, miR-196b, lnc-p21, miR-100, and lnc-OIP5-AS1 can strengthen the radiosensitivity of gastrointestinal carcinoma cells by altering DNA damage repair and promoting irradiation-induced apoptosis by binding to their respective target genes (Bandres et al., 2009; Wang et al., 2014; Zhang et al., 2014; Yang et al., 2015; Lin et al., 2016; Afshar et al., 2018; Luo et al., 2018; Ruhl et al., 2018; Shen et al., 2018; Zou et al., 2018; Chen et al., 2019; Samadi et al., 2019; Ge et al., 2020). Furthermore, several reports indicated that the characteristics of tumor cell stemness and EMT profoundly influenced the sensitivity of tumor cells to RT. miR-130a sensitized RC cells to RT by targeting SOX4 and inhibiting transcription of the EMT-related genes and NBS1 (Ha Thi et al., 2019). miR-15b enhanced the radiosensitivity of CRC cells by interacting with DCLK1 to inhibit the EMT via regulating BMI1 and β‐catenin expression (Ji et al., 2018). miR-124 and miR-145 boosted the radiosensitivity of CRC cells by inhibiting the cell stemness by targeting PRRX1 (Zhang et al., 2014; Lin et al., 2016; Zhu et al., 2018). In addition, some studies found that increasing G2/M phase arrest could significantly improve the radiosensitivity of tumor cells. For instance, lnc-p21, let-7e, miR-196b, and miR-320 improved the radiosensitivity of cells by blocking cell cycle transition via the Wnt/β-catenin pathway (Wang et al., 2014; Wan et al., 2015; Shen et al., 2018; Chen et al., 2019; Samadi et al., 2019). circ-CBL.11 was increased in CRC cells after irradiation and elevated circ-CBL.11 reinforced the radiosensitivity by repressing the phosphorylation of P53 through sponging to miR-6778-5p to regulate the YWHAE expression (Li et al., 2019b). In addition, miR-214 expression was downregulated after exposure to irradiation both in CRC cells and plasma of CRC patients. Mechanistically, miR-214 enhanced the radiosensitivity by suppressing cell autophagy through LC3 repression and elevating P62 via directly binding to ATG12 (Hu et al., 2018). miR-21-5p targeted SATB1 in SW480 cells to improve the sensitivity to RT (Lopes-Ramos et al., 2014). Zhang et al. revealed that CREB increased miR-630 expression by binding in the promoter region of miR-630; in turn, miR-630 regulated the radiosensitivity of CRC cells by targeting BCL2L2 and TP53RK (Zhang et al., 2015). Lnc-NEAT1 was also upregulated in CRC cells after irradiation. Elevated lnc-NEAT1 enhanced the GSDME-mediated pyroptosis resulting in the radiosensitivity of CRC cells by competitively binding to miR-448 (Su et al., 2021). Thus, ncRNAs could be used as therapeutic targets in RT by exploring the molecular mechanism of radiosensitivity (Figure 1).

### Non-Coding RNAs Induce Radioresistance

Several studies have reported a close relationship between ncRNAs and radioresistance of RT in cancer therapy (Fan et al., 2018; Zhang et al., 2019b; Zheng et al., 2020). As shown in Table 2, Chen et al. (2020) investigated that miR-93-5p was upregulated in CRC tissues and induced the resistance to RT in CRC cells by facilitating cell growth and suppressing irradiation-induced apoptosis. Lnc-00152, miR-155, and miR-222 were elevated in the radioresistant CRC cell lines. Reduced levels of lnc-00152 in radioresistant cells significantly repressed the migratory and invasiveness of CRC cells (Chen et al., 2018). miR-155 and miR-222 induced the radioresistance in CRC cells by promoting cell proliferation and DNA damage repair (Khoshinani et al., 2017). Moreover, after miR-21 upregulation, the radioresistant characteristics of CC cells were enhanced by promoting cell cycle transition and cell invasion and inhibiting irradiation-induced apoptosis (Deng et al., 2014). Gao et al. (2021) reported that circ_0055625 was increased in CC tissues and cell lines, and overexpressed circ_0055625 significantly reduced the sensitivity of CC cells to RT. Besides, Liu et al. (2020a) found that lnc-RI significantly interfered with sensitivity of CRC cells to RT by improving the cell viability and DNA damage repair and preventing irradiation-induced apoptosis. Lnc-HOTAIR was also increased in CRC tissues, cell lines, and serum of patients after RT. Furthermore, lnc-HOTAIR resulted in radioresistance by promoting cell growth and cell autophagy and restraining irradiation-induced apoptosis *in vitro* and *in vivo* (Yang et al., 2016; Liu et al., 2020b). The resistance of CRC cells to RT was induced *in vitro* after miR-622 upregulation in the cells (Ma et al., 2015). Overexpression of miR-224 reduced the sensitivity of CRC cells to RT *in vitro* (Salendo et al., 2013). Elevated miR-210 resulted in radioresistance in CC cells, as miR-210 enhanced cell survival and autophagy (Sun et al., 2015). Increased miR-29a levels caused the resistance of CRC cells and intestinal cells to irradiation (Wang et al., 2016). Circ-CCDC66 was also increased in radioresistant CC tissues compared to radiosensitive tissues and induced the resistance of CC cells to irradiation by boosting cell growth and constraining irradiation-induced apoptosis (Wang et al., 2019b). Xiao et al. (2020) demonstrated that lnc-TRPM2-AS promoted the resistance of GC cells to RT by improving cell survival fraction and promoting DNA damage repair. Circ-ABCB10, circ-BANP, lnc-ROR, and miR-183-5p were also elevated in CRC tissues and cell lines. Circ-ABCB10 resulted in radioresistance of CRC cells by facilitating cell growth and promoting EMT (Xie et al., 2021a). Circ-BANP reduced the sensitivity of CRC cells to irradiation by elevating cell survival fraction and stimulating cell autophagy (Xie et al., 2021b). Moreover, the inhibition of lnc-ROR alleviated the resistance of CRC cells to RT by constraining cell growth and boosting irradiation-induced apoptosis (Yang et al., 2017). Furthermore, miR-183-5p exacerbated the resistance of CRC cells to RT, increasing cell survival and stimulating cell proliferation *in vitro* and *in vivo* (Zheng et al., 2019). Lnc-UCA1 was increased in CRC tissues, CC cell lines, and tissues from RT patients. Lnc-UCA1 interfered with the radiosensitivity of CRC cells by boosting EMT and G2/M arrest and suppressing irradiation-induced apoptosis (Yang et al., 2018). Yu et al. (2021) reported that lnc-TLCD2-1 was downregulated in CRC tissues and radiosensitive cell lines. lnc-TLCD2-1 induced the radioresistant status of CRC cells by elevating cell viability and repressing irradiation-induced apoptosis. miR-106b was increased in CRC tissues and highly differentiated CRC cell lines. Overexpressed miR-106b conferred radioresistance to CRC cells by facilitating tumor-initiating capacity, cell survival fraction, and DNA damage repair (Zheng et al., 2015). Zhang et al. (2021) found that LINC00909 was increased in tissue samples from LARC patients that did not respond to neoadjuvant chemoradiotherapy. Furthermore, overexpression of LINC00909 induced cell resistance to RT *in vivo* and *in vitro*. Lnc-EGOT was upregulated in RC tissues and cell lines. Lnc-EGOT significantly facilitated cell growth and inhibited the irradiation-induced apoptosis of RC cells, thereby resulting in cell resistance to RT (Li et al., 2021b). Thus, ncRNAs play critical roles in the resistance of gastrointestinal carcinoma to irradiation.

**TABLE 2:** The radioresistance induction of non-coding RNAs in gastrointestinal carcinoma.

Cancer type	Non-coding RNAs	Expression	Sources	Sample number	Targets	Biological functions	Upstream	References
CRC	miR-93-5p	Increased	Tissue	75 paired	FOXA1	Facilitate cell proliferation, inhibit radiation-induced apoptosis, and promote radiation resistance	EVs	Chen et al. (2020)
CC	Circ_0055625	Increased	Tissue and cell line	57 paired	miR-338-3p/MSI1	Facilitate cell proliferation, migration, and invasion, repress radiation-induced Apoptosis, and induce radiation resistance	Irradiation	Gao et al. (2021)
CRC	miR-224	—	—	—	—	Induce radiation resistance	—	Salendo et al. (2013)
CRC	LINC00152	Increased	Cell line	—	—	Facilitate cell proliferation, migration, and invasion, and promote radiation resistance	—	Chen et al. (2018)
CRC	miR-155 and miR-222	Increased	Cell line	—	—	Facilitate cell proliferation and induce radiation resistance	Irradiation	Khoshinani et al. (2017)
CC	miR-21	—	—	—	hMSH2	Inhibit irradiation-induced apoptosis, enhance cell growth, invasion, cell cycle transition, and induce radiation resistance	—	Deng et al. (2014)
CRC	lnc-RI	—	—	—	miR-4727-5p/LIG4	Facilitate cell growth and cell cycle transition, repress radiation-induced apoptosis, and induce radiation resistance	—	Liu et al. (2020a)
CRC	lnc-HOTAIR	Increased	Serum, tissue and cell line	12 paired +71 paired	MiR-93/ATG12	Facilitate cell viability and cell autophagy, repress radiation-induced cell apoptosis, and induce radiation resistance	—	Liu et al. (2020b)
CRC	lnc-HOTAIR	Increased	Tissue and cell line	53 paired	—	Promote cell proliferation, migration, and invasion, inhibit radiation-induced apoptosis, and induce radiation resistance	—	Yang et al. (2016)
RC	miR-622	Increased	Tissue and cell line	17	RB1	Increase surviving fraction and induce radiation resistance	Irradiation	Ma et al. (2015)
CC	miR-210	—	—	—	Bcl-2	Increase cell growth and autophagy, inhibit radiation-induced apoptosis, and induce radiation resistance	HIF-1α	Sun et al. (2015)
CRC	miR-29a	Increased	Cell line	—	PTEN	Increase surviving fraction and induce radiation resistance	Irradiation	Wang et al. (2016)
CC	circ-CCDC66	Increased	Tissue and cell line	84	miR-338-3p	Increase cell viability and surviving fraction, and induce radiation resistance	Irradiation	Wang et al. (2019b)
GC	lnc-TRPM2-AS	Increased	Tissue and cell line	80 paired	miR-612/IGF2BP1 and FOXM1	Increase survival fractions and DNA damage repair, and induce radiation resistance	Irradiation	Xiao et al. (2020)
CRC	circ-ABCB10	Increased	Tissue and cell line	20 paired	miR-217	Promote cell proliferation, migration, invasion, and induce radiation resistance	—	Xie et al. (2021a)
CRC	circ-BANP	Increased	Tissue and cell line	20 paired	miR-338-3p	Increase cell viability, cell survival fraction and cell autophagy, and induce radiation resistance	—	Xie et al. (2021b)
CRC	lnc-ROR	Increased	Tissue and cell line	30 paired	p53/miR-145	Promote cell viability, inhibit radiation-induced apoptosis, and induce radiation resistance	—	Yang et al. (2017)
CRC	miR-183-5p	Increased	Tissue and cell line	39 paired	ATG5	Enhance cell viability and survival fraction, and induce radiation resistance	—	Zheng et al. (2019)
CRC	lnc-UCA1	Increased	Tissue and cell line	32 paired	—	Promote cell proliferation, cell cycle transition and EMT, inhibit radiation-induced apoptosis, and induce radiation resistance	—	Yang et al. (2018)
CRC	lnc-TLCD2-1	Decreased	Tissue and cell line	10 paired	miR-193a-5p/YY1	Promote cell proliferation, inhibit radiation-induced apoptosis, and induce radiation resistance	-	Yu et al. (2021)
CRC	miR-106b	Increased	Tissue and cell line	15 paired	PTEN and p21	Enhance the tumor-initiating cell capacity, cell survival fraction and DNA damage repair, and induce radiation resistance	—	Zheng et al. (2015)
LARC	LINC00909	Increased	Tissue	31	—	Enhance cell viability and induce radiation resistance	—	Zhang et al. (2021)
RC	Lnc-EGOT	Increased	Tissue and cell line	40 paired	miR-211-5p/ErbB4	Promote cell proliferation, inhibit radiation-induced apoptosis, and induce radiation resistance	Irradiation	Li et al. (2021b)

CRC, colorectal cancer; EVs, extracellular vesicles; CC, colon cancer; GC, gastric cancer; RC, rectal cancer; EMT, epithelial-mesenchymal transition.

### The Mechanism of Radioresistance Induction

**FIGURE 2:**
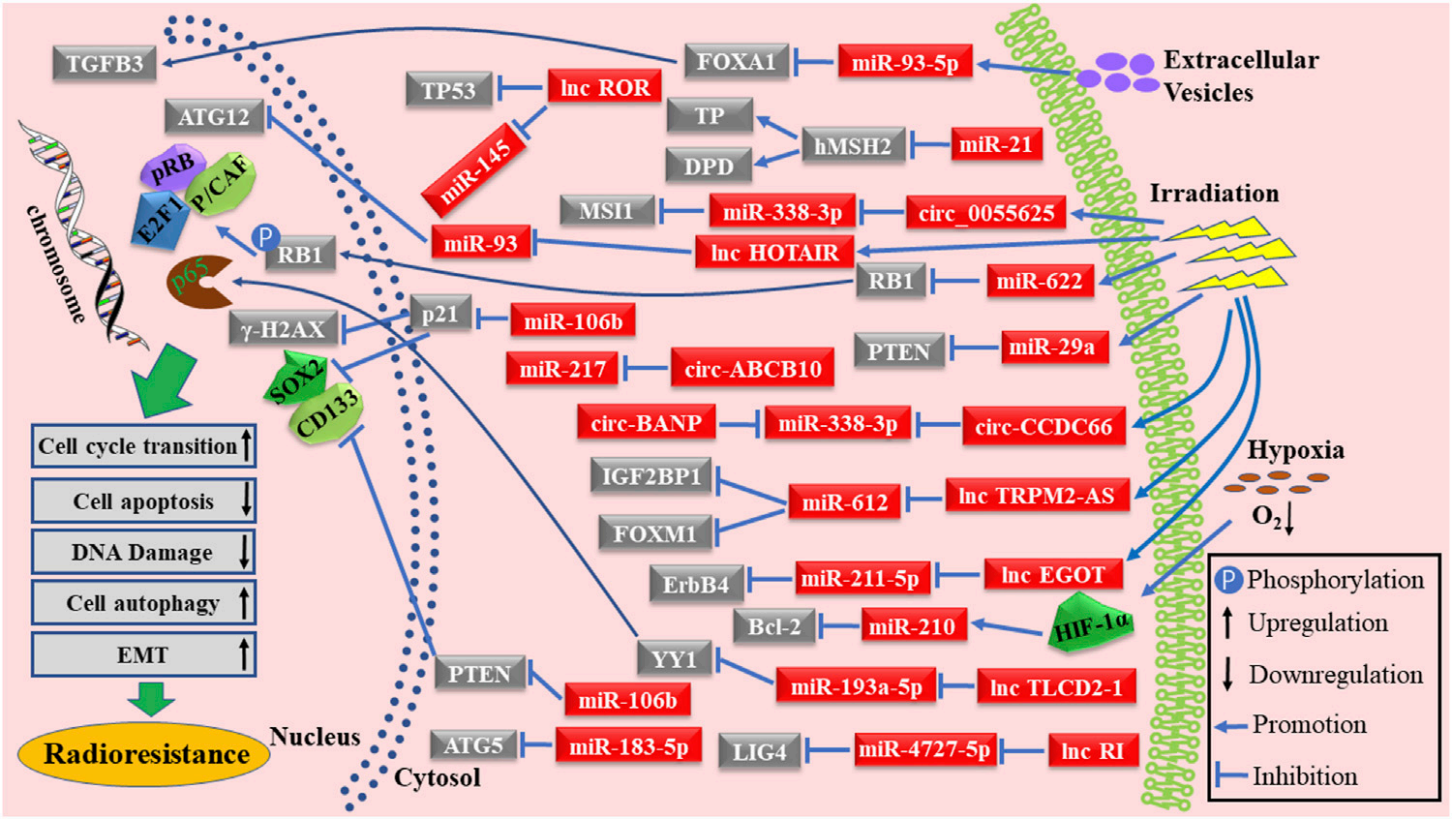
Schematic diagram of ncRNAs as radioresistance inducers in gastrointestinal carcinoma.

RT has been widely accepted as an essential treatment for various cancers and it is the recommended treatment strategy for patients with locally advanced gastrointestinal carcinoma (LAGC) (Bhide and Nutting, 2010; Cercek et al., 2018; Hajj and Goodman, 2015). Unfortunately, resistance to RT is becoming widespread, currently being one of the main limitations of RT treatment (Jin et al., 2020; Pera et al., 2012). Therefore, understanding the molecular mechanisms leading to radioresistance, as well as conducting clinical translational therapies, may significantly improve the prognosis of patients with gastrointestinal carcinoma. The mechanisms of ncRNAs in radioresistance induction are presented in Figure 2. Cancer-associated fibroblasts (CAF)-derived extracellular vesicles can deliver miR-93-5p to induce radioresistance in CRC cells by targeting FOXA1 and elevating TGFB3 via the TGF-β signaling pathway (Chen et al., 2020). miR-21 also conferred radioresistance to CC cells by binding to hMSH2 mRNA and modulating TP and DPD expression (Deng et al., 2014). Circ-005625, lnc-HOTAIR, miR-622, miR-29a, circ-CCDC66, lnc-TRPM2-AS, and lnc-EGOT were upregulated in gastrointestinal carcinoma cells after irradiation. Elevated circ-005625 conferred the radioresistant ability to CC cells by regulating MSI1 expression via sponging miR-338-3p (Gao et al., 2021). lnc-HOTAIR acted as a molecular sponge of miR-93 to regulate ATG12-mediated autophagy in CRC cells, resulting in radioresistance (Liu et al., 2020b). miR-622 regulated the activity of the p-Rb-E2F1-P/CAF complex to affect the radioresistance of CRC cells by binding to RB1 (Ma et al., 2015). miR-29a activated the PI3K/Akt pathway to cause radioresistance in CRC cells and intestinal cells by directly targeting PTEN (Wang et al., 2016). Both circ-CCDC66 and circ-BANP induced the resistance of CC cells and CRC cells to irradiation by competitively binding to miR-338-3p (Wang et al., 2019b; Xie et al., 2021b). Lnc-TRPM2-AS resulted in the resistance of GC cells to irradiation via modulating the IGF2BP1 and FOXM1 expression by sponging miR-612 (Xiao et al., 2020). Lnc-EGOT conferred the resistance to RT in RC cells via accumulating ErbB4 by sponging miR-211-5p (Li et al., 2021b). Lnc-RI conferred radioresistance to CRC cells by adjusting LIG4 expression via binding to the miR-4727-5p (Liu et al., 2020a). HIF-1α is an important protein increased in hypoxic conditions; Sun et al. reported that miR-210 levels increased with the elevation of HIF-1α and caused the radioresistance of CC cells through targeting the Bcl-2 (Sun et al., 2015). Circ-ABCB10 targeted miR-217 to cause radioresistance in CRC cells (Xie et al., 2021a). Yang et al. revealed that lnc-ROR induced radioresistance in CRC cells by inhibiting the translation of P53 and reducing miR-145 (Yang et al., 2017). miR-183-5p abated the response of CRC cells to RT by directly binding to ATG5 (Zheng et al., 2019). Lnc-TLCD2-1 activated the NF-кB pathway to cause radioresistance in CRC cells via regulating YY1 by targeting miR-193a-5p (Yu et al., 2021). Increased miR-106b levels downregulated PTEN and p21 expression and subsequently enhanced radioresistance in CRC cells (Zheng et al., 2015). Insights into the mechanisms of radioresistance induction might provide therapeutic orientation for patients with gastrointestinal carcinoma.

### Non-Coding RNAs Predict the Response to RT

ncRNAs have been widely investigated as diagnostic biomarkers or therapeutic efficacy predictors in cancer (Li et al., 2020a; Ha Thi et al., 2021; Konoshenko et al., 2021). Accurate prediction of response to RT by ncRNAs profiles would undoubtedly improve the prognosis of LAGC patients and allow individualized treatment. Azizian et al. reported that low expression of miR-573 in the tissues of patients with RC showed a better response to RT (Azizian et al., 2016). RC patients with lower levels of circulating miR-18b and miR-20a presented a better outcome to preoperative RT. Notably, miR-18b and miR-20a showed high specificity and sensitivity to distinguish those patients with negative postoperative nodal stage after RT (Azizian et al., 2015). Furthermore, the positive predictive value (PPV) and negative predictive value (NPV) of miR-18b and miR-20a were 0.35 and 0.79, 0.41, and 0.85, respectively. miR-200c was decreased in LARC patients with advanced T-stage. In addition, downregulated miR-200c was closely related to non-responsive primary or recurrent LARC to neoadjuvant RT (Bhangu et al., 2014). miR-21, miR-99b, and miR-375 were greatly decreased in RC patients with better tumor regression after preoperative RT. The area under the curve (AUC) value of the combination of miR-21, miR-99b, and miR-375 was 0.736 (sensitivity of 0.60; specificity of 0.829) to distinguish RC patients with better response from others (Campayo et al., 2018). LARC patients with high miR-21 expression predicted a good response to preoperative RT. The AUC value of miR-21 was 0.736 with a sensitivity and specificity value of 0.866 and 0.60, respectively (PPV = 0.92 and NPV = 0.428), to distinguish patients with a complete response from those with a non-complete response (Caramés et al., 2015). LARC patients with a high level of miR-31 expression predicted a poor response to preoperative RT. The AUC value of the miR-31 was 0.71 with 0.608 sensitivity and 0.763 specificity (PPV = 0.518 and NPV = 0.823) to discriminate between LARC patients with minimal, moderate, complete, or no response (Caramés et al., 2016). LARC patients with a high miR-125b in tissues or serum predicted a poor response to preoperative RT. The AUC of miR-125b in tissue and plasma was 0.9026 and 0.7821 to separate RC patients that did not respond from those that did, respectively (D’Angelo et al., 2016). miR-194 was increased in LARC tissues of patients responding to RT, and elevated miR-194 predicted a good outcome for neoadjuvant RT (D’Angelo et al., 2018). miR-1183, miR-483-5p, miR-622, miR-125a-3p, miR-1224-5p, miR-188-5p, miR-1471, miR-671-5p, miR-1909, miR-630, and miR-765 were greatly increased in the LARC tissues of patients that achieved pathological complete response (pCR), while miR-1274b and miR-720 were decreased in the LARC tissues of good response patients after neoadjuvant RT. In addition, miR-622 and miR-630 had 100% sensitivity and 100% specificity in dividing patients with pCR from non-response patients (Della Vittoria Scarpati et al., 2012). Drebber et al. demonstrated that miR-145 was increased in post-therapeutic tissues compared to pre-therapeutic specimens of LARC patients, and a low level of post-therapeutic miR-145 expression presented a poor response to neoadjuvant RT (Drebber et al., 2011). Du et al. revealed that miR-548c-5p, miR-548d-5p, and miR-663a were upregulated in patients with pCR compared to non-complete response patients and an elevated cluster of microRNAs indicated a good response to RT of RC patients (Du et al., 2018; Du et al., 2019). Ji et al. (2018) discovered that miR-15b was greatly reduced in CRC tissues compared to adjacent normal tissues, and elevated miR-15b predicted a good outcome after neoadjuvant RT. miR-31 and miR-30c were greatly reduced in the serum of RC patients compared to healthy controls. In addition, miR-31 and miR-30c were also decreased in the serum of patients after the completion of neoadjuvant RT and radical surgery (Jo et al., 2017). miR-451a, miR-502–5p, miR-223–3p, and miR-1246 were increased in the partial responders compared to non-responders via microarray analysis. Furthermore, higher miR-451a expression was confirmed in the serum of complete responders compared to that of non-responders and partial responders (Kelley et al., 2017). Through microarray analysis, miR-16, miR-590-5p, miR-153, miR-519c-3p, and miR-561 were upregulated in the tissues of RC responders. miR-16, miR-590-5p, and miR-153 were used to distinguish complete responders from incomplete responders with 100% accuracy. miR-519c-3p and miR-561 were used to discriminate between good responders and poor responders with 100% predictive power (Kheirelseid et al., 2013). Li et al. discovered a close connection between lncRNA-miRNA-mRNA regulation network and the response of LARC patients to neoadjuvant RT (Li et al., 2019c). Although lnc-p21 was downregulated in CRC tissues, its expression was increased in the tissues and serums of responders. RC patients with a high level of lnc-p21 expression in tissues also showed a good response to postoperative RT (Li et al., 2020c). Circulating miR-506-3p and miR-140-5p were upregulated in the plasma of radiosensitive CRC patients, and patients with a high level of miR-506-3p and miR-140-5p in the serum exhibited a good response to RT. The predictive accuracy of miR-506-3p and miR-140-5p was 0.925 to separate radiosensitive patients from radioresistant patients (Liao et al., 2020). miR-214 was overexpressed in radiosensitive CRC specimens, while its expression in plasma decreased in CRC patients after RT. Moreover, a higher expression of miR-214 in tissues predicted a better response to RT for CRC patients (Hu et al., 2018). The upregulated miR-21-5p was validated in the tissues of RC responders via microarray analysis and qPCR. Higher miR-21-5p expression correlated with a better response to RT. The sensitivity and specificity of miR-21-5p in discriminating good outcomes from RC patients to RT were 0.78 and 0.86, respectively (Lopes-Ramos et al., 2014). miR-519b-3p was also overexpressed in the tissues of LARC responders. The AUC value of miR-519b-3p was 0.91 with 100% sensitivity and 0.81 specificity in distinguishing responsive and non-responsive patients (Luo et al., 2018). Increased miR-622 expression was found in non-regression tumors of patients with RC. Besides, higher miR-622 expression predicted a worse outcome for RT (Ma et al., 2015). Elevated miR-487a-3p expression was confirmed in the tissues of non-responder LARC patients using multi-phase verifications. The AUC value of the miR-487a-3p was 0.766 with 0.78 sensitivity and 0.60 specificity to distinguish patients with non-response from response (Machackova et al., 2020). Millino et al. reported that miR-630 was upregulated in the tissues of RC non-responders and decreased in the tissues of responders (Millino et al., 2017). Additionally, Ruhl et al. demonstrated that RC patients with a partial response to RT frequently expressed high levels of miR-451a in tissues (Ruhl et al., 2018). Salendo et al. (2013) found that 14 microRNAs were increased, and 22 microRNAs were decreased in the radioresistant CRC cell lines via microarray analysis. Higher levels of miR-125b and miR-137 expression in the tissues of RC patients usually determined a worse response to RT (Svoboda et al., 2008). High expression of let-7e, miR-196b, miR-450a, miR-450b-5p, and miR-99a predicted a good response to RT in LARC patients, while high expression of miR-215, miR190b, and miR-29b-2 predicted a poor response. Using these miRNAs, the PPV and NPV are 0.9 and 0.9 to distinguish responders from non-responders (Svoboda et al., 2012). Xiong et al. reported that three circRNAs and one lncRNA were increased and two circRNAs and five lnc-RNAs were decreased in the radioresistant CRC cell lines *via* microarray analysis and qRT-PCR (Xiong et al., 2015; Xiong et al., 2017). Xu et al. (2014) also revealed that lnc-R05532, lnc-NR_015441, and lnc-NR_033374 were positively correlated with the resistance of CRC cell lines to irradiation. By using microarray analysis and qRT-PCR, elevated miR-345 expression was confirmed in the tissues and plasma of non-responder LARC to RT. In addition, LARC patients with high expression of miR-345 in tissues or serum usually faced a poor response to RT. The AUC value of the plasmatic miR-345 was 0.75 to distinguish patients with a response from non-response (Yu et al., 2016). LARC patients with low expression of DBET, LINC00909, and FLJ33534 often showed a poor response to RT. The accuracy of DBET, LINC00909, and FLJ33534 was 0.65, 0.82, and 0.67, respectively, to differentiate LARC patients between response and non-response (Zhang et al., 2021). As shown in Table 3, ncRNAs can effectively predict the outcome of gastrointestinal carcinoma patients to RT.

**TABLE 3:** The predictive response of radiotherapy of non-coding RNAs in gastrointestinal carcinoma.

Cancer type	Non-coding RNAs	Expression in responder	Sources	Sample number	Predictive value	References
RC	miR-18b and miR-20a	Low	Serum	42	Patient with reduced expression of miR-18b (specificity: 0.50, sensitivity: 0.67, PPV = 0.35, NPV = 0.79) and miR-20a (specificity: 0.57, sensitivity: 0.75, PPV = 0.41, NPV = 0.85) during CRT was associated with negative postoperative nodal stage	Azizian et al. (2015)
RC	miR-21, miR-99b and miR-375 combination	Low	Tissue	96	Patient with low expression of miR-21, miR-99b, and miR-375 combination shows a good response to CRT. The AUC value of the combination of three miRNAs was 0.736 with 0.60 sensitivity and 0.829 specificity to distinguish patients with maximum response from others	Campayo et al. (2018)
LARC	miR-125b	Low	Serum and tissue	34 and 38	Patient with high expression of miR-125b in serum or tissue shows a poor response to CRT. The AUC value of the miR-125b in tissue was 0.9026 to distinguish patients with non-response from response. The AUC of circulating miR-125b is 0.7821 to distinguish patients with non-response from response	D’Angelo et al. (2016)
LARC	miR-451a	High	Tissue and Serum	45 + 45	Patient with high expression of miR-451a in serum or tissue shows a good response to RT.	Kelley et al. (2017)
LARC	miR-15b	High	Tissue	92	Patient with high expression of miR-15b shows a good response to CRT.	Ji et al. (2018)
CRC	miR-506-3p and miR-140-5p	High	Serum	18	Patient with high expression of miR-506-3p and miR-140-5p shows a good response to RT. The AUC value of the miR-506-3p and miR-140-5p was 0.925 to distinguish patients with radiosensitive from radioresistant	Liao et al. (2020)
CRC	miR-214	High	Tissue and serum	42 + 10	Patient with high expression of miR-214 in tissue shows a good response to RT.	Hu et al. (2018)
RC	miR-21-5p	High	Tissue	43	Patient with high expression of miR-21-5p shows a good response to CRT. Overall sensitivity and specificity of miR-21-5p in predicting complete response to CRT was 0.78 and 0.86, respectively	Lopes-Ramos et al. (2014)
LARC	miR-519b-3p	High	Tissue	55	Patient with high expression of miR-519b-3p shows a good response to CRT. The AUC value of the miR-519b-3p was 0.91 with 100% sensitivity and 81% specificity to distinguish patients with response from non-response	Luo et al. (2018)
RC	miR-451a	High	Tissue	12	Patient with high expression of miR-451a shows a good response to RT.	Ruhl et al. (2018)
LARC	miR-622	Low	Tissue	17	Patient with high expression of miR-622 shows a poor response to RT.	Ma et al. (2015)
LARC	DBET, LINC00909 and FLJ33534	Low	Tissue	89	Patient with high expression of DBET, LINC00909 and FLJ33534 in tissue shows a poor response to neoadjuvant CRT. The AUC value of the DBET, LINC00909 and FLJ33534 in tissue was 0.65, 0.82, and 0.67, respectively, to distinguish patients with response from non-response	Zhang et al. (2021)
RC	miR-573	Low	Tissue	147	Patient with low expression of miR-573 shows a good response to CRT.	Azizian et al. (2016)
RC	miR-200c	High	Tissue	69	Patient with low miR-200c is associated with non-response in primary tumors and recurrent cancers to neoadjuvant RT.	Bhangu et al. (2014)
LARC	miR-21	High	Tissue	92	Patient with high expression of miR-21 shows a good response to CRT. The AUC value of the miR-21 was 0.736 with 0.866 sensitivity and 0.60 specificity (PPV = 0.92, NPV = 0.428) to distinguish patients with complete response from noncomplete response	Caramés et al. (2015)
LARC	miR-31	Low	Tissue	78	Patient with high expression of miR-31 shows a poor response to CRT. The AUC value of the miR-31 was 0.71 with 0.608 sensitivity and 0.763 specificity (PPV = 0.518, NPV = 0.823) to distinguish patients with non-response from response	Caramés et al. (2016)
LARC	miR-194	High	Tissue	38 + 29	Patient with high expression of miR-194 shows a good response to CRT.	D’Angelo et al. (2018)
RC	miR-1183, 483-5p, 622, 125a-3p, 1224-5p, 188-5p, 1471, 671-5p, 1909, 630, 765, 1274b, 720	High (miR-1183, 483-5p, 622, 125a-3p, 1224-5p, 188-5p, 1471, 671-5p, 1909, 630, 765) and low (miR-1274b, 720)	Tissue	38	Patient with high expression of miR-1183, 483-5p, 622, 125a-3p, 1224-5p, 188-5p, 1471, 671-5p, 1909, 630, 765 shows a good response to CRT. Patient with low expression of miR-1274b and miR-720 shows a good response to CRT. miR-622 and miR-630 had a 100% sensitivity and specificity in selecting pathological complete response cases	Della Vittoria Scarpati et al. (2012)
LARC	miR-145	High	Tissue	40	Patient with low intratumoral post-therapeutic expression of miR-145 shows a poor response to CRT.	Drebber et al. (2011)
LARC	miR-548c-5p, miR-548d-5p, and miR-663a	High	Tissue	38	Patient with high expression of miR-548c-5p, miR-548d-5p, and miR-663a shows a good response to CRT.	Du et al. (2019)
RC	miR-16, miR-590-5p, miR-153, miR-519c-3p, miR-561	High	Tissue	12	Three miRNA transcripts (miR-16, miR-590-5p, and miR-153) to predict complete versus incomplete response and two miRNA transcripts (miR-519c-3p and miR-561) to predict good versus poor response with a median accuracy of 100%	Kheirelseid et al. (2013)
CRC	lnc-p21	High	Tissue and serum	177 + 20	RC patient with high expression of lnc-p21 in tissue shows a good response to post-operative CRT.	Li et al. (2020c)
LARC	miR-487a-3p	Low	Tissue	87	Patient with high expression of miR-487a-3p shows a poor response to CRT. The AUC value of the miR-487a-3p was 0.766 with 0.78 sensitivity and 0.60 specificity to distinguish patients with non-response from response	Machackova et al. (2020)
LARC	miR-630	Low	Tissue	59	Patient with high expression of miR-630 shows a poor response to CRT.	Millino et al. (2017)
RC	miR-125b and miR-137	Low	Tissue	66	Patient with high expression of miR-125b and miR-137 shows a poor response to CRT.	Svoboda et al. (2008)
LARC	miR-215, 190b, 29b-2, 196b, 450a, 450b-5p, 99a and let-7e	High (let-7e, miR-196b, 450a, 450b-5p, 99a) and low (miR-215, 190b and miR-29b-2)	Tissue	20	Patient with high expression of let-7e, miR-196b, miR-450a, miR-450b-5p, and miR-99a shows a good response to CRT. Patient with high expression of miR-215, miR190b, and miR-29b-2 shows a poor response to CRT. Using these miRNAs, the PPV and NPV are 0.9 and 0.9 to distinguish patients with response from non-response	Svoboda et al. (2012)
LARC	miR-345	Low	Tissue and Serum	20 + 129	Patient with high expression of miR-345 in tissue or serum shows a poor response to CRT. The AUC value of the miR-345 in serum was 0.75 to distinguish patients with response from non-response	Yu et al. (2016)

RC, rectal cancer; CRT, chemoradiotherapy; PPV, positive predictive value; NPV, negative predictive value; AUC, area under the curve; LARC, locally advanced rectal cancer; RT, radiotherapy; CRC, colorectal cancer.

### Clinical Application of Non-coding RNAs

Despite their potential, the use of ncRNAs for therapy poses the following limitations *in vivo*: poor cellular uptake, unstable pharmacological structures, off-target effects, and possible immunogenicity (Singh et al., 2018). However, it is still possible to manipulate these molecules for cancer therapy, combined with the effective application of RNA-delivering systems, such as chemical modifications of ncRNAs, lipid-based ncRNAs delivery systems, and organic/inorganic nanoparticles (Rupaimoole and Slack, 2017; Singh et al., 2018). In addition, aberrant profiles of ncRNAs in the tumor tissues or the circulation can also be used to predict the long-term survival of patients (Flippot et al., 2019; Yuan et al., 2020; Sharma et al., 2021). Resorting effective treatments would undoubtedly and significantly improve the outcome of patients with LAGC before tumor progression (Deng et al., 2019; Tomita et al., 2020; Wang et al., 2020; Sun et al., 2021). Bandres et al. demonstrated that not only GC patients with stage III but also the whole GC patients with lower expression of miR-451 predicted shorter disease-free survival (DFS) and overall survival (OS) (Bandres et al., 2009). The expression of miR-15b was negatively connected with the adverse clinicopathological characteristics and liver metastasis of CRC patients. In addition, patients with low miR-15b were significantly associated with worse therapeutic results of neoadjuvant therapy and poor DFS and OS (Ji et al., 2018). The survival would be significantly shortened in CC patients with a high level of circ_0055625 expression (Gao et al., 2021). Liu et al. reported that the expression of lnc-HOTAIR was negatively correlated to the survival of CRC patients via the analysis of follow-up data (Liu et al., 2020b). Xiao et al. (2020) revealed that the high lnc-TRPM2-AS expression was accurately forecasted advanced clinicopathological characteristics and significantly correlated to the shorter OS and recurrence-free survival (RFS) of GC patients. Meanwhile, high lnc-TLCD2-1 expression predicted worse OS and disease-specific survival of CRC patients from the GSE17536 dataset (Yu et al., 2021). For CRC patients with high miR-183-5p expression, the OS was worse (Zheng et al., 2019). High miR-573 or low miR-200c usually predicted poor OS and cancer-specific survival of RC patients (Bhangu et al., 2014; Azizian et al., 2016). Campayo et al. reported that the low level of miR-21, miR-99b, and miR-375 combination was correlated to a worse DFS in RC patients (*p* = 0.068) (Campayo et al., 2018). High miR-31 indicated poor OS in LARC patients (Caramés et al., 2016). Li et al. (2020c) reported various predictive roles of lnc-p21 in CRC patients. High lnc-p21 levels determined poor OS and DFS in CRC or RC patients. For RC patients who underwent postoperative CRT, high lnc-p21 meant better OS. High levels of lnc-p21 in the plasma of CRC patients also suggested a worse OS. Low plasmatic miR-345 usually signified better 3-year local RFS for LARC patients (Yu et al., 2016). Low DBET and LINC00909 often suggested a better OS in patients. However, high DBET, LINC00909, and FLJ33534 usually indicated a poor DFS in patients with CRC (Zhang et al., 2021). The abovementioned data validated the critical role and clinical value of ncRNAs as prognostic biomarkers in gastrointestinal carcinoma (Table 4).

**TABLE 4:** The clinical application of non-coding RNAs in gastrointestinal carcinoma.

Cancer type	Non-coding RNAs	Expression	Sources	Sample number	Prognosis	References
CC	circ_0055625	Increased	Tissue	57	Worse survival of CC patients with high circ_0055625	Gao et al. (2021)
RC	miR-21, miR-99b and miR-375 combination	Low in responder	Tissue	96	Mean DFS for patients with low levels were 74.5 months, while it was 78.8 months for those with high levels (*p* = 0.068)	Campayo et al. (2018)
GC	miR-451	Decreased	Tissue	67 (45 for Kaplan-Meier analysis)	Shorter DFS and OS for patients with low miR-451	Bandres et al. (2009)
CRC	miR-15b	Decreased	Tissue	135	Shorter DFS and OS for patients with low miR-15b	Ji et al. (2018)
CRC	lnc-HOTAIR	Increased	Serum/Tissue	12/71	Poor prognosis of CRC patients with high lnc-HOTAIR	Liu et al. (2020b)
GC	lnc-TRPM2-AS	Increased	Tissue	80	Worse OS and RFS for GC patients with high lnc-TRPM2-AS	Xiao et al. (2020)
CRC	miR-183-5p	Increased	Tissue	39	Worse OS for CRC patients with high miR-183-5p	Zheng et al. (2019)
CRC	lnc-TLCD2-1	Decreased	Tissue	10	Worse OS and DSS for CRC patients with high lnc-TLCD2-1	Yu et al. (2021)
CRC	DBET, LINC00909and FLJ33534	Low in responder	Tissue	138	Low expression of DBET and LINC00909 was associated with a better DFS and OS in CRC patients. High expression of the FLJ33534 was associated with a worse DFS in CRC patients	Zhang et al. (2021)
RC	miR-573	Low in responder	Tissue	147	Worse OS and CSS for patient with high miR-573	Azizian et al. (2016)
RC	miR-200c	High in responder	Tissue	69	Worse OS and CSS for patient with low miR-200c	Bhangu et al. (2014)
LARC	miR-31	Low in responder	Tissue	78	Worse OS for patient with high miR-31	Caramés et al. (2016)
CRC	lnc-p21	High in responder	Tissue/Serum	177/20	Worse OS and DFS for CRC or RC patient with high lnc-p21. Better OS for RC patient with high lnc-p21 from post-operative CRT. Worse OS for CRC patient with high plasmatic lnc-p21 from mesenteric vein	Li et al. (2020c)
LARC	miR-345	Low in responder	Tissue/Serum	20/129	Better 3-year local recurrence free survival for patient with low plasmatic miR-345	Yu et al. (2016)

GC, gastric cancer; CRC, colorectal cancer; LARC, locally advanced rectal cancer; RC, rectal cancer; DFS, disease-free survival; OS, overall survival; RFS, recurrence free survival; DSS, disease-specific survival; CSS, cancer-specific survival; PFS, progression-free survival.

## Conclusion

RT has been used in the clinic to treat patients with localized advanced gastrointestinal carcinomas. The use of RT directly leads to DNA damage, mainly caused by double-strand breaks in tumor cells. RT also indirectly damages tumor cells through the activation of downstream genes. ncRNAs can act as radiosensitivity enhancers or radioresistance inducers in gastrointestinal carcinoma by affecting DNA damage repair, cell cycle arrest, irradiation-induced apoptosis, cell autophagy, stemness, EMT, and cell pyroptosis through targeting various genes (Figures 1, 2). In addition, the predictive value of ncRNAs in response to RT was evaluated. ncRNAs could be used to guide individualized treatments. Overall, further studies are needed to explore the potential value of ncRNAs in RT and gastrointestinal carcinoma.

## Abbreviations

CC, colon cancer; CRC, colorectal cancer; DFS, disease-free survival; EMT, epithelial-mesenchymal transition; GC, gastric cancer; LARC, locally advanced rectal cancer; OS, overall survival; RC, rectal cancer; RFS, recurrence-free survival; RT, radiotherapy; TME, tumor microenvironment.
